# Accurate Sequence-Based Prediction of Deleterious nsSNPs with Multiple Sequence Profiles and Putative Binding Residues

**DOI:** 10.3390/biom11091337

**Published:** 2021-09-09

**Authors:** Ruiyang Song, Baixin Cao, Zhenling Peng, Christopher J. Oldfield, Lukasz Kurgan, Ka-Chun Wong, Jianyi Yang

**Affiliations:** 1 School of Mathematical Sciences, Nankai University, Tianjin 300071, China; ruiyang@mail.nankai.edu.cn (R.S.); caobaixin@mail.nankai.edu.cn (B.C.); 2 Research Center for Mathematics and Interdisciplinary Sciences, Shandong University, Qingdao 266237, China; zhenling@email.sdu.edu.cn; 3 Department of Computer Science, Virginia Commonwealth University, Richmond, VA 23284, USA; cjoldfield@vcu.edu; 4 Department of Computer Science, City University of Hong Kong, Kowloon Tong, Hong Kong SAR, China; kc.w@cityu.edu.hk

**Keywords:** mutation, sequence profile, binding site

## Abstract

Non-synonymous single nucleotide polymorphisms (nsSNPs) may result in pathogenic changes that are associated with human diseases. Accurate prediction of these deleterious nsSNPs is in high demand. The existing predictors of deleterious nsSNPs secure modest levels of predictive performance, leaving room for improvements. We propose a new sequence-based predictor, DMBS, which addresses the need to improve the predictive quality. The design of DMBS relies on the observation that the deleterious mutations are likely to occur at the highly conserved and functionally important positions in the protein sequence. Correspondingly, we introduce two innovative components. First, we improve the estimates of the conservation computed from the multiple sequence profiles based on two complementary databases and two complementary alignment algorithms. Second, we utilize putative annotations of functional/binding residues produced by two state-of-the-art sequence-based methods. These inputs are processed by a random forests model that provides favorable predictive performance when empirically compared against five other machine-learning algorithms. Empirical results on four benchmark datasets reveal that DMBS achieves AUC > 0.94, outperforming current methods, including protein structure-based approaches. In particular, DMBS secures AUC = 0.97 for the SNPdbe and ExoVar datasets, compared to AUC = 0.70 and 0.88, respectively, that were obtained by the best available methods. Further tests on the independent HumVar dataset shows that our method significantly outperforms the state-of-the-art method SNPdryad. We conclude that DMBS provides accurate predictions that can effectively guide wet-lab experiments in a high-throughput manner.

## 1. Introduction

Non-synonymous single-nucleotide polymorphisms (nsSNPs) are mutations in the DNA sequences that result in the mutations of amino acids in the corresponding protein sequences. While some nsSNPs may not be deleterious, others may destabilize protein structure and/or protein-ligand interactions leading to loss of function. These deleterious nsSNPs are associated with a range of diseases [1,2,3,4]. For example, sickle cell anemia (SKCA), which is caused by the variant of glutamate to valine at position 7 of the hemoglobin subunit beta (UniProt id: P68871), can lead to microvascular occlusion, thus cutting off the blood supply to nearby tissues. While the effects of mutations can be studied experimentally, such analysis is often limited to a small subset of possible mutations given the time- and cost-expensive nature of these studies. Computational analysis provides a cost- and time-efficient alternative to pre-select the most-promising mutations for the wet lab analysis [5,6,7].

A substantial amount of research was devoted to developing predictors of deleterious nsSNPs. Several SNP-related databases have been established, including dbSNP [8], SNPdbe [9], and dbNSFP [10]. Those resources make it possible to conceptualize, design, and empirically test and compare the computational predictors. SIFT is the first algorithm that was designed to predict deleterious nsSNPs [11]. SIFT relies on the presumption that mutations of highly conserved amino acids tend to be deleterious. Several follow-up studies were done to design predictors that improve the predictive accuracy, such as (in chronological order) SNAP [12], PolyPhen-2 [13,14], Logit [15], MutationTaster2 [16], SNPdryad [17], PROVEAN [18], and EVmutation [19]. These methods have advanced the predictive models primarily by using more sophisticated information that they extract from the input protein chains. PolyPhen-2 considers sequence conservation computed from the multiple sequence alignment (MSA) as well as features extracted from the corresponding protein structure, if available. MutationTaster2 relies on pre-computed conservation scores generated with BLAST and access to several mutation databases. SNPdryad improves the computation of conservation by constructing MSA using orthologous rather than homologous sequences and by utilizing several conservation scoring schemes. PROVEAN relies on datasets of pre-computed conservation scores and categorizes residues into several classes (substitutions, insertions, deletions, nonsense mutations, and frameshifts). Finally, EVmutation further improves computation of the conservation by considering dependencies between positions using evolutionary coupling. Moreover, the current tools utilize rather simple predictive models, such as Bayesian classifiers (PolyPhen-2 and MutationTaster2), logistic regression (Logit), and scoring functions (PROVEAN and EVmutation).

We aim to further improve the predictive performance by implementing two innovations. First, we develop a new approach to quantify the conservation by constructing and combining multiple MSAs generated with multiple sequence databases and by using two complimentary alignment tools. Second, we utilize information about putative ligand-binding residues that we predict in the input protein sequences with recently developed bioinformatics methods. Mutations at these positions may disrupt these binding events, consequently leading to the loss of function without disrupting the protein structure. Finally, we empirically test and compare several machine-learning algorithms and we select the one that generates models characterized by the highest levels of predictive performance. Benchmarks on several test datasets reveal that our model outperforms the current methods.

## 2. Materials and Methods

### 2.1. Overview of the Proposed Method

**Figure 1:**
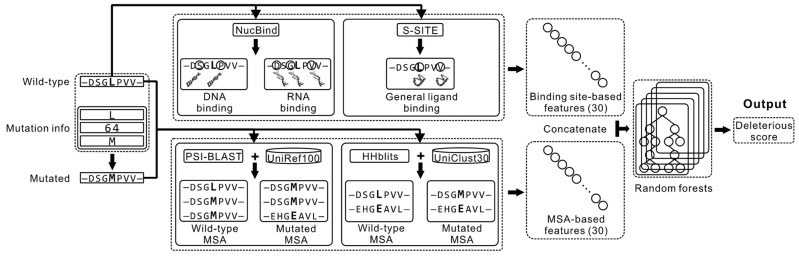
The architecture of the proposed DMBS method. The inputs to DMBS include the wild-type sequence and the mutation information (wild-type residue, mutated position and mutated residue). The wild-type sequence is submitted to two sequence-based binding site prediction methods, NucBind and S-SITE, to predict DNA/RNA and general ligand binding sites, respectively. Meanwhile, both the wild-type and the mutated sequences are searched against two sequence databases (UniRef100 and UniClust30) with two algorithms (PSI-BLAST and HHblits, respectively) to construct four MSA profiles. A total of 60 features are extracted from the predicted binding site information and the MSA profiles, which are fed into the random forests algorithm to predict the deleterious score of the mutation.

Figure 1 summarizes the architecture of the proposed DMBS method. The input is the wild-type sequence and the mutation information. We process the wild-type sequence with the NucBind [20] and S-SITE [21] methods to predict ligand-binding residues. More specifically, NucBind is a machine-learning-based algorithm that predicts nucleic acids-binding residues in the input protein sequence. S-SITE is a template-based algorithm that utilizes sequence profile-profile alignment to accurately predict residues that interact with a non-specific (generic) ligand in the given protein chain. Residues that are predicted by these methods are likely to be functionally important as they facilitate the protein-ligand interactions, and thus their mutations could lead to the loss of function. In parallel, we feed the wild-type and the mutated sequences into two complementary alignment programs, the sequence-sequence alignment-based PSI-BLAST [22,23] and the HMM (hidden Markov model) profile-HMM profile alignment-based HHblits [24]. PSI-BLAST performs alignment against the UniRef100 database while HHblits runs alignment against the UniClust30 databases to construct the two corresponding MSAs. Next, we use the putative binding residues produced by the two predictors and the two MSAs to extract a fixed-size vector of numeric features. The conversion into the fixed-size vector is necessary since this is required by the subsequently used machine-learning models, which are unable to process the variable length MSA and predictions that depend on the input sequence length. Finally, we process these features by a machine-learning model to predict the propensity for deleterious nsSNPs. We empirically compare the quality of results generated by six different machine-learning algorithms (see Section 3.2) and we select the best-performing algorithm, random forests, for our DMBS predictor.

### 2.2. Feature Extraction

We convert the two MSAs profiles and the two predictions of the ligand-binding residues into fixed-size vector of numeric features as detailed follows.

#### 2.2.1. Features Computed from the MSA Profiles

To perform the conversion, first we use the two MSAs to compute the corresponding position-specific probability matrices (PSPMs). The PSPM has *L* × 20 size where *L* is the number of amino acids in the protein sequence and 20 represents the 20 amino acid types. The PSPM’s *p_i,j_* element represents the likelihood of occurrence of the *j*-th amino acid type at the *i*-th column of the MSA (i.e., the *i*-th position in the protein sequence) generated by a given alignment algorithm. The entropy at each position *i* is then calculated.

(1) $$e_{i}=-\sum_{j=1}^{20}p_{i,j}\log p_{i,j}\;,1\leq i\leq L$$

We compute the entropy at the mutated position *k*, *e_k_*, the z-score of *e_k_*, as well as the mean *μ* and standard deviation *σ* of the entropy values generated with a given alignment tool over the wild-type and the mutated sequences as follows (8 features):

(2) $$z_{k}=\frac{e_{k}-\mu }{\sigma }$$

(3) $$\mu =\frac{1}{L}\sum_{i=1}^{L}e_{i}$$

(4) $$\sigma =\sqrt{\frac{1}{L-1}\sum_{i=1}^{L}{\left(e_{i}-\mu \right)}^{2}}$$

We also calculate the difference between the corresponding four features for the wild-type and the mutated sequences (4 features). Moreover, we compute the inner product between the *k*-th row of the PSPM matrix generated by a given alignment method and the corresponding row of the BLOSUM62 matrix at position *k* as follows

(5) $$b_{k}=\sum_{j=1}^{20}p_{k,j}B(A_{k},j)\;$$

where *A_k_* is the amino acid type at the *k*-th position of the input sequence (wild-type or mutated); the index *j* varies through the 20 standard amino acids; *B*(*A_k_*, *j*) is the corresponding element in the BLOSUM62 matrix. We compute the inner product *b_k_* for the wild-type chain, the mutated sequence, and we also compute their difference (3 features).

Consequently, we convert the MSAs for the wild-type and the mutated chains generated by the two alignment methods (PSI-BLAST and HHblits) into the set of 2 × (8 + 4 + 3) = 30 features.

#### 2.2.2. Features Computed from the Sequence-Based Prediction of the Ligand-Binding Residues

We use NucBind to produce predictions of the DNA-binding and RNA-binding residues and S-SITE to predict the generic ligand-binding residues. We extract features by screening these predictions with the conservation computed from the PSI-BLAST-generated MSA. We also tried HHblits’ MSA, but it did not provide further improvements in the predictive performance.

Suppose that *m* binding residues were predicted by the two predictors. First, we compute conservation of these *m* positions using three diverse approaches. We use the entropy Equation (1), the inner product Equation (5), and the Kullback-Leibler (KL) divergence, which is defined as follows:

(6) $$d_{\mathrm{KL}}(P_{i}||Q_{i})=\sum_{j=1}^{20}p_{i,j}\log\frac{p_{i,j}}{q_{i,j}}\;,1\leq i\leq L$$

where *P_i_* and *Q_i_* are two vectors representing the *i*-th row of the PSPM matrices of the wild-type and mutated sequences, respectively.

Second, we identify the position in the sequence with the highest absolute difference in the conservation score between the wild-type and mutated chains. We do this for each of the three conservation measures. The delta in the conservation values serves as a proxy for propensity of function destabilization. Third, we compute three sets of features: (1) The value of the highest absolute difference in conservation; (2) the linear distance (along the sequence) of the position identified in step 2 to the mutated position; and (3) the propensity for ligand binding generated by a given predictor for the position identified in step 2. We calculate three features for each of the three conservation scores and each of the three predictors of binding residues for the total of 3 (features) × 3 (conservation measures) × 3 (predictors) = 27 features (note that NucBind predicts DNA and RNA binding residues, respectively, and we consider it as two predictors here). Moreover, we compute the linear distance (along the sequence) between the mutated position and the closest predicted binding residue for each of the three predictors, which results in 3 features. In total, we compute 27 + 3 = 30 features using the sequence-based prediction of the ligand-binding residues.

We combine the 30 features generated from MSA and the 30 features produced with the help of the ligand-binding residues predictors to obtain a 60-dimensional feature vector that we use to make prediction of the deleterious nsSNPs.

### 2.3. Benchmark Datasets

We use a comprehensive collection of four benchmark datasets that were developed in past studies to evaluate and compare our method with the current tools. The first two are HumDiv and HumVar (version 2.1.0) downloaded from the PolyPhen-2 Web site [13]. The other two are SNPdbe [9] and ExoVar [15]. These datasets have been widely used to evaluate predictors of deleterious nsSNPs [11,13,17,19]. Table 1 summarizes these datasets.

### 2.4. Performance Evaluation

The predictors of the deleterious nsSNPs generate two types of outputs: The binary value (deleterious vs. non-deleterious mutation) and numeric propensities representing the likelihood that a given mutation is deleterious. We assess the quality of the numeric propensities using a commonly used metric, the Area Under the receiver operating characteristic Curve (AUC). AUC values range between 0.5 and 1 with higher values denoting more accurate predictions. We evaluate the binary predictions using accuracy (rate of correct predictions) and Matthew’s correlation coefficient (MCC). Like other correlation coefficients, MCC ranges between −1 and 1 where 0 corresponds to random-level predictive quality (no correlation between the binary predictions and the native annotations) and a larger positive value denotes a more accurate prediction (stronger correlation). We test predictive performance using the 10-fold cross validation on each of the four benchmark datasets and conduct a set of independent tests on the largest HumVar dataset.

## 3. Results and Discussion

### 3.1. Deleterious nsSNPs Coincide with the Location of the Putative Ligand-Binding Residues

**Figure 2:**
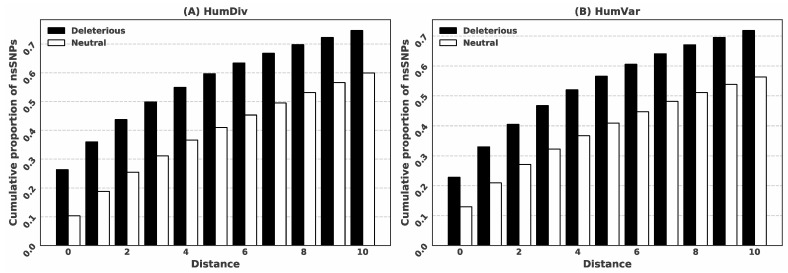
Cumulative proportion of the predicted ligand-binding positions for the deleterious and neutral nsSNPs. The distance is the linear distance (along the sequence) between a given nsSNP position and the position of the closest predicted binding residue.

We investigate whether the locations of the predicted binding residues are useful to predict deleterious nsSNPs using the HumDiv and HumVar datasets. We assert that a given biologically important position in the protein sequence, defined by being predicted to interact with ligands, is unlikely to mutate during evolution. In other words, mutations at such positions are likely to cause deleterious effects. We compare the rates of the predicted ligand-binding residues among the positions that have deleterious vs. non-deleterious nsSNPs to test this assertion. We predict residue-binding 4786 times among the 20,985 deleterious mutations in the HumVar dataset, (4786/20,985 = 22.81% rate, compared to 2742 out of the 21,138 non-deleterious mutations (12.97% rate). The difference reveals that the prediction of the binding residues provides useful clues to identify deleterious nsSNPs. In addition, the mutated position does not have to directly overlap with the prediction of ligand-binding to result in the loss of function, but instead they could be in close proximity in the sequence. Therefore, we consider the linear distance between the mutated position and the closest position of the putative binding residues as a useful predictive input (see Section 2.2). Figure 2 shows the two cumulative ratios (for deleterious vs. non-deleterious mutations) for distances ranging between 0 and 10 for both datasets. The figure reveals that the ratios for the deleterious mutations are consistently higher than the ratios for the neutral mutations across the entire range of distances. This analysis justifies our decision to include the prediction of the ligand-binding residues as one of the key inputs for our predictive model.

### 3.2. Comparison of Predictive Performance Generated Using Different Machine Learning Algorithms

We consider six machine-learning algorithms to produce the predictive models. They include random forests (RF), naive Bayes (NB), logistic regression (LR), multilayer perceptron (MLP), adaptive boosting that uses decision tree as the base predictor (Ada), and extreme gradient boosting (XGB). We use the implementations from the scikit-learn package with their default parameters [25].

**Figure 3:**
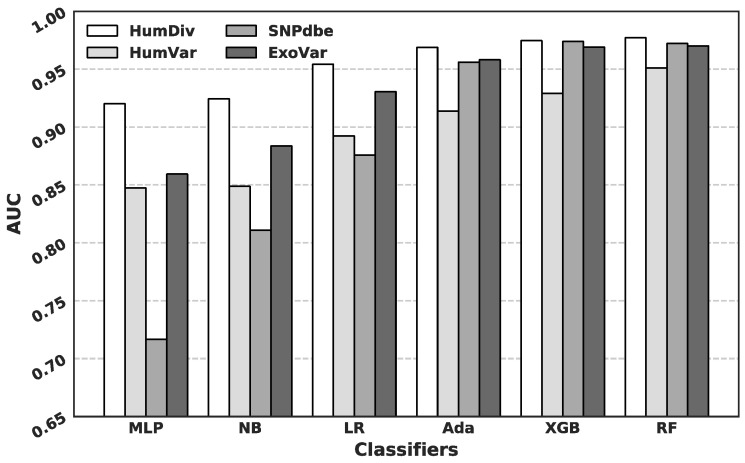
AUCs for the predictions generated by the six considered machine-learning algorithms on the four benchmark datasets.

The AUC values produced by each of the six algorithms for the four benchmark datasets are summarized in Figure 3. The figure reveals that the RF model provides the highest AUCs across the four datasets, with the second best being the XGB model that underperforms for the HumVar dataset. More detailed comparisons can be found in Supplementary Table S1. Moreover, the training time for the second- and third-best algorithms (XGB and Ada, respectively) is much longer than the runtime needed to train the RF model. The favorable predictive performance of RF is in agreement with a similar study performed for the SNPdryad method [17]. The authors of that study compared seven machine-learning algorithms (RF, NB, Bayesian network, MLP, Ada, support vector machine, and k-nearest neighbor) on the HumDiv and HumVar datasets. Using different implementations and different predictive inputs, they also conclude that RF outperforms the other machine-learning algorithms. To compare, other recent predictors of deleterious mutations utilize much less sophisticated predictive models, such as the Bayesian model [13,14,16], logistic regression [15], and simple scoring functions [18,19]. Consequently, given the prior success of this algorithm and our empirical results, we select the RF model for our DMBS predictor.

### 3.3. Comparison with Current Predictors on the HumDiv and the HumVar Datasets

We compare DMBS with three state-of-the-art predictors, SIFT, PolyPhen2, and SNPdryad, on the HumDiv and the HumVar datasets. Table 2 shows the AUC values while the accuracy and MCC values for DMBS are reported in Supplementary Table S1; the corresponding metric are not available for the other predictors. Table 2 reveals that the AUCs for all methods on the HumDiv dataset are relatively high (>0.9). Two predictors, DMBS and SNPdryad, achieve the same highest values of AUC = 0.98. The third-best predictor, PolyPhen2, secures a much lower AUC = 0.95. The DMBS’s AUC on the HumVar dataset is 0.95, which is substantially higher than AUCs of the other three methods, with the second-best SNPdryad securing AUC = 0.91. We note that the difference by 0.04 is equivalent to an 8% improvement given that the AUC ranges between 0.5 and 1.

We introduce two innovations that arguably drive the improvements over the current tools: The use of predicted binding residues and the combination of MSAs computed with two complementary alignment programs and sequence databases. We perform an ablation study to investigate the impact of these innovations on the predictive performance of our model. We compute two reduced versions of our model, DMBS_BR and DMBS_MSA, by training the RF model with a subset of features extracted from the predicted binding residues and from the MSAs alone, respectively. We compare these two variants with the complete DMBS model in Table 2. We observe that AUCs of the two variants are relatively high (i.e., comparable or better than the AUCs of the current methods) and lower than the AUC of DMBS, suggesting that both types of inputs have high predictive power and provide complementary information. The DMBS_MSA variant outperforms DMBS_BR on both datasets. This is expected as the MSA-derived conservation provides a more “generic” predictive input that covers both structural (structural stability) and functional (stability of protein-ligand interactions) aspects of the underlying sequence, while the use of the putative binding residues targets only the latter aspect. Moreover, DMBS_MSA achieves an AUC similar to SNPdryad on the HumDiv dataset and outperforms SNPdryad on the HumVar dataset. This suggests that the use of the complementary alignment programs and sequence databases that are explored in DMBS_MSA provides stronger predictive input than the use of a single alignment program that considers orthologous sequences, as it was done in SNPdryad that utilizes the same RF model.

We evaluate the significance of the differences between the best-performing DMBS, its two variants, and the best current method, SNPdryad (Supplementary Table S2). The differences between AUCs of DMBS and the other three approaches on the HumVar dataset are significant (*p*-value < 0.01). For the HumDiv dataset, the AUCs of DMBS and SNPdryad are not statistically different while DMBS is significantly better than its two variants (*p*-value < 0.01). Overall, we conclude that DMBS outperforms the three current methods (SIFT, PolyPhen2, and SNPdryad) when tested across two benchmark datasets, and we argue that these improvements stem from the use of two complementary and useful types of predictive inputs.

**Figure 4:**
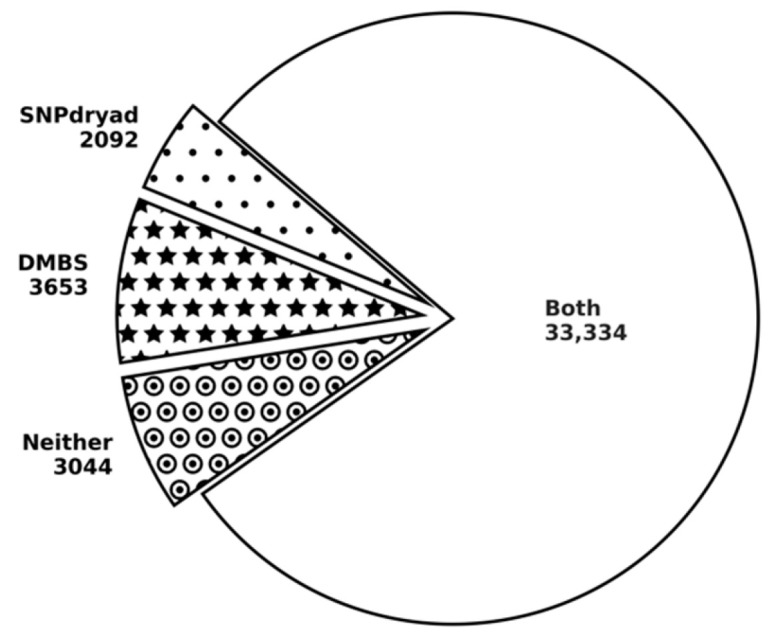
Comparison of the predictions by DMBS and SNPdryad on the HumVar dataset.

We provide a more detailed side-by-side comparison between DMBS and SNPdryad, the best-performing of the three current methods tested here, on the HumVar dataset. We calibrate the binary predictions for both methods by using cutoffs that that equalize the false positive and false negative rates. In other words, predictions with propensities above a given cutoff are assumed to be deleterious mutations. These thresholds equal 0.505 and 0.515 for DMBS and SNPdryad, respectively. We compare the two sets of putative deleterious nsSNPs in Figure 4. We find that a significant majority of the nsSNPs (33,334 nsSNPs) is predicted correctly by both methods, compared to 3044 nsSNPs for which both methods fail (marked by circle-dots). DMBS correctly predicts 3653 deleterious nsSNPs that are incorrectly predicted by SNPdryad (marked by stars), compared to a smaller number of 2092 mutations that SNPdryad predicts successfully and for which DMBS fails (marked by dots). The bottom line is that DMBS secures a modest improvement over SNPdryad (3653 vs. 2092 correct predictions).

### 3.4. Comparison with Current Predictors on the SNPdbe and ExoVar Datasets

We assess DMBS on two additional benchmark datasets, SNPdbe and ExoVar, and compare DMBS’s results with six current predictors: SNPdryad, SNAP, PolyPhen2, SIFT, MutationTaster, and Logit. We were unable to test this extended group of methods on the other two datasets (HumDiv and the HumVar) since SNAP, MutationTaster, and Logit did not report results on these benchmark sets.

**Figure 5:**
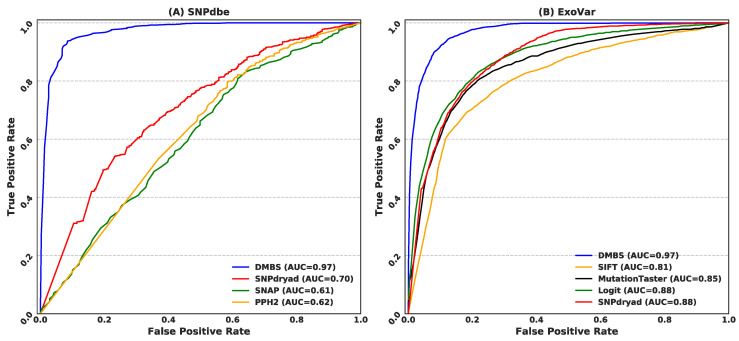
ROC curves and the corresponding AUC values for DMBS and six state-of-the-art predictors on the SNPdbe dataset (**A**) and the ExoVar datasets (**B**). The ROC curves for the six predictors were adopted from the work SNPdryad.

Figure 5 shows the AUC values and the corresponding ROC curves for DMBS and the other six methods on the two datasets. Figure 5A suggests that DMBS outperforms the other three methods that were tested on the SNPdbe dataset by a large margin. DMBS’s AUC on the SNPdbe dataset equals 0.97 compared to the AUC of 0.70 for the second-best SNPdryad predictor. We note that DMBS’s AUC is consistent with the results on the HumDiv and HumVar datasets (Table 2). The large margin of the improvement over the current methods on this dataset prompted us to investigate the possible reason for the high predictive quality of our predictive model. To this end, we remove the new features that we introduce in DMBS (features based on the predicted binding residues and the HHblits-based alignment), rebuild the predictive models, and re-test this version of our predictor on the SNPdbe dataset. The AUC of this reduced model is 0.70, which is virtually the same as the AUC of SNPdryad. This reveals that the substantial improvement that DMBS offers is due to the use of the novel predictive inputs.

We compare the results on the ExoVar dataset in Figure 5B. DMBS secures AUC = 0.97 on this dataset, outperforming the second-best SNPdryad that obtains AUC = 0.88. Once again, the AUC of DMBS is consistent with the results on the other three benchmark datasets (SNPdbe, HumDiv and HumVar). The AUC of the other methods, including SIFT, MutationTaster, and Logit, ranges between 0.81 and 0.88.

Supplementary Table S2 compares the predictive performance of DMBS against its two variants, DMBS_MSA and DMBS_BR, which are described in Section 3.3. We show that the full implementation of DMBS provides significantly better AUC values than its two reduced variants (*p*-value < 0.05) across both datasets, SNPdbe and ExoVar. This is consistent with the results on the HumDiv and the HumVar datasets.

In summary, our empirical assessment that relies on four diverse benchmark sets shows that DMBS consistently achieves high levels of predictive performance (AUC ≥ 0.95). It outperforms several representative current predictors when compared side-by-side across these datasets. We also demonstrate that these improvements can be attributed to the use of the innovative predictive inputs.

### 3.5. Comparison with SNPdryad Based on Independent Test

The above comparisons with SNPdryad were all based on 10-fold cross validation, which has been used by most methods for deleterious nsSNPs prediction. However, an independent test is usually needed to ensure an unbiased evaluation. To this end, rather than performing 10-fold cross validation, we randomly split the largest dataset HumVar into two subsets: 80% for training and the remaining 20% for independent testing. For each split, we retrained DMBS and SNPdryad using the training set and evaluated their performance on the test set.

**Figure 6:**
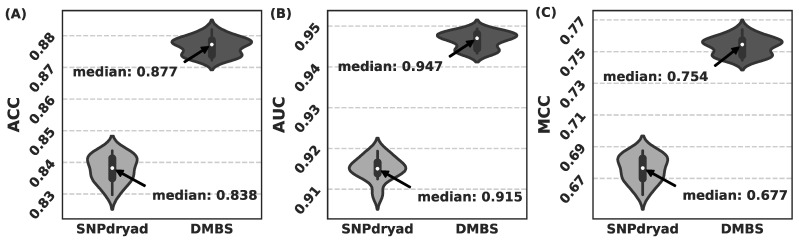
Comparison between DMBS and SNPdryad based on 20 repeated independent tests on the HumVar dataset. The results of DMBS and SNPdryad are represented by violin plots, based on ACC (**A**), AUC (**B**), and MCC (**C**), with each plot showing the distribution, median, and range of the corresponding metric values.

The results of 20 repeated independent tests are shown in Figure 6. In the 20 independent tests, we compare the median value for each metrics, and DMBS’s ACC, AUC, and MCC are 0.877, 0.947, and 0.754, respectively, outperforming SNPdryad on all three metrics (with *p*-value < 1 × 10^−30^ for all the three metrics).

### 3.6. DMBS Web Server

To facilitate the use a DMBS, we set up a web server at https://yanglab.nankai.edu.cn/DMBS/ (accessed on 9 August 2021). The input to the server includes the FASTA-formatted protein sequence, the mutation position, and the mutated residue type. Though trained for the prediction of deleterious nsSNPs, DMBS can be applied to any types of residue mutations. Thus, users are allowed to specify up to 19 residue types for mutation. An option is provided to keep the users’ data private. In general, the predictions can be completed in about 30 min for a typical protein of 300 residues. The results are accessible by a prediction-specific URL, which is assigned upon job submission. The major results include the predicted secondary structure, disordered information, and deleterious scores for the specified mutations, which are visualized in a figure and listed in a table as well.

## 4. Conclusions

Accurate prediction of the deleterious nsSNPs is needed to guide wet-lab mutational studies. While several tools that predict those mutations are available, further improvements in the predictive performance are possible and still desired. We aim to provide these improvements via the use of innovative predictive modeling.

We introduced DMBS, a modern data science algorithm that provides accurate predictions of deleterious nsSNPs with model interpretability. The method works by combining two types of inputs, sequence conservation and predicted ligand-binding residues. We generate the sequence conservation scores using a comprehensive set of MSA profiles produced by two diverse alignment tools and two different protein databases. We extract information concerning the putative ligand-binding residues from two state-of-the-art sequence-based methods. We use four benchmark datasets to demonstrate that DMBS secures accurate and robust levels of predictive performance, with AUC ranging between 0.95 and 0.98 across the considered datasets. We also show that DMBS improves six state-of-the-art predictors when compared side-by-side on these datasets. Finally, we perform ablation analysis that suggests that the improvements offered by DMBS stem from the use of the novel interpretable inputs.

## Figures and Tables

**Table 1:** Summary of the benchmark datasets used in this work.

Dataset	Deleterious nsSNPs	Neutral nsSNPs	Ratio of Positives	Reference
HumVar	20,985	21,138	49.82%	[13]
HumDiv	5322	7070	42.95%	[13]
SNPdbe	2263	307	88.05%	[9]
ExoVar	4567	3145	59.22%	[15]

**Table 2:** AUCs for DMBS, two the DMBS variants (DMBS_BR and DMBS_MSA), and three current predictors of deleterious nsSNPs on the HumDiv and HumVar benchmark datasets. The AUCs were calculated based on the 10-fold cross validation. The highest AUC values for each dataset are highlighted in bold font. Summary of the benchmark datasets used in this work.

Method	HumDiv	HumVar
SIFT	0.91	0.86
PolyPhen2	0.95	0.89
SNPdryad	**0.98**	0.91
DMBS_BR	0.908	0.909
DMBS_MSA	0.976	0.941
DMBS	**0.977**	**0.948**

## Data Availability

The data presented in this study are available in [13] (HumVar and HumDiv) [9] (SNPdbe), and [15] (ExoVar).

## Supplementary Materials

The following are available online at https://www.mdpi.com/article/10.3390/biom11091337/s1, Table S1: Evaluation results of considered classifiers on four benchmark datasets, Table S2: Assessment of the statistical significance.
