# Supplementary material for: Accurate Sequence-Based Prediction of Deleterious nsSNPs with Multiple Sequence Profiles and Putative Binding Residues
Source: Biomolecules. 2021 Sep 9;11(9):1337. doi: 10.3390/biom11091337 (PMC8469993; doi:10.3390/biom11091337)
Supplement: Supplementary file 1 [file biomolecules-11-01337-s001.zip › biomolecules-1354666-supplementary.pdf]

Supplementary Materials

Accurate sequence-based prediction of deleterious nsSNPs with multiple sequence profiles and putative binding residues

Ruiyang Song [1†], Baixin Cao [1†], Zhenling Peng [2], Christopher J Oldfield [3], Lukasz Kurgan [3, *], Ka-Chun Wong [4], and Jianyi Yang [1, *]

Table S1. Accuracy, Area Under the ROC Curve (AUC), Matthews correlation coefficient (MCC), True Positive Rate (TPR), and False Negative Rate (FPR) of six machine learning algorithms on the four benchmark datasets.

Methods	Accuracy				AUC				MCC				TPR				FPR			
	HumVar	HumDiv	SNPdbe	ExoVar	HumVar	HumDiv	SNPdbe	ExoVar	HumVar	HumDiv	SNPdbe	ExoVar	HumVar	HumDiv	SNPdbe	ExoVar	HumVar	HumDiv	SNPdbe	ExoVar
MLP[1]	0.7853	0.8608	0.8440	0.8102	0.8475	0.9203	0.7167	0.8594	0.5708	0.7149	0.3015	0.6104	0.7742	0.8008	0.9019	0.8233	0.2036	0.0941	0.5831	0.2089
NB[2]	0.7730	0.8725	0.5451	0.8102	0.8488	0.9243	0.8108	0.8837	0.5531	0.7420	0.2377	0.6030	0.8514	0.8756	0.5020	0.8739	0.3049	0.1298	0.1368	0.2824
LR[3]	0.8204	0.8952	0.8957	0.8763	0.8924	0.9543	0.8757	0.9306	0.6421	0.7871	0.3551	0.7431	0.8512	0.8923	0.9872	0.9034	0.2102	0.1027	0.7785	0.1631
Ada[4]	0.8410	0.9127	0.9447	0.8968	0.9137	0.9689	0.9560	0.9581	0.682	0.8219	0.7264	0.7853	0.8472	0.8991	0.9757	0.9339	0.1652	0.0771	0.2834	0.1571
XGB[5]	0.8562	0.9229	0.9502	0.9085	0.9292	0.9746	0.9739	0.9690	0.7123	0.8428	0.7514	0.8098	0.8572	0.9121	0.9806	0.9464	0.1449	0.0689	0.2736	0.1466
RF[6]	0.8815	0.9279	0.9518	0.9113	0.9481	0.9771	0.9722	0.9700	0.7630	0.8527	0.7528	0.8159	0.8780	0.9051	0.9890	0.9507	0.1150	0.0549	0.3225	0.1459

[1]Multilayer Perceptron (MLP); [2]Naïve Bayes (NB); [3]Logistic Regression (LR); [4]Adaptive Boosting (Ada); [5]eXtreme Gradient Boosting (XGB); [6]Random Forests (RF, also as the DMBS predictor).

We implement the above algorithms based on the scikit-learn package with their default parameters [1].

Table S2. Assessment of the statistical significance of the differences between AUC values produced by DMBS and AUCs of several other predictors including two DMBS variants, DMBS_MSA and DMBS_BR (described in Section 3.3 in the main text), and SNPdryad on the four benchmark datasets (HumDiv, HumVar, SNPdbe and ExoVar). The table lists *p*-values that were computed using student's *t*-tests.

Method	HumDiv	HumVar	SNPdbe	ExoVar
DMBS_MSA	8.47E-12	9.72E-50	8.05E-20	9.14E-23
DMBS_BR	4.05E-74	2.65E-70	0.02	2.07E-13
SNPdryad	1	2.12E-75	--	--

References

1. Pedregosa F, Varoquaux G, Gramfort A et al. Scikit-learn: Machine Learning in Python, Journal of Machine Learning Research 2011;12:2825-2830.
